# Supplementary material for: Streptococcus suis infection on European farms is associated with an altered tonsil microbiome and resistome
Source: Microb Genom. 2024 Dec 19;10(12):001334. doi: 10.1099/mgen.0.001334 (PMC11657566; doi:10.1099/mgen.0.001334)
Supplement: Uncited Supplementary Material 1. [file mgen-10-01334-s001.pdf]

Supplementary Materials Files

Supplementary information on farms.

This document contains information on farms relevant to understanding the farm practice and disease situation. The information provided is qualitative and based on information from veterinarians and farm workers. The study farms were selected based on previous contact and represent a wide range of *S. suis* problem scenarios and are not intended to be representative of the different countries. The Spanish farms were sampled during severe outbreaks with a subset of symptomatic piglets sampled, while German and Dutch farms were sampled during smaller outbreaks with all available case animals being sampled.

Table 1. Farm overview.

Farm	Country	Symptoms	Disease stage	Farm disease history
DE1	Germany	Meningitis	Post-weaning	Yes
DE2	Germany	None	None	None
DE3	Germany	Meningitis	Post-weaning	Yes
DE4	Germany	Meningitis	Post-weaning	Yes
DE5	Germany	Meningitis	Post-weaning	Yes
DE6	Germany	Meningitis+Arthritis	Post-weaning	Yes
ES1	Spain	None	None	None
ES2	Spain	Meningitis+Arthritis	Post-weaning	Yes
ES3	Spain	Arthritis	Pre-weaning	Yes
ES4	Spain	Arthritis	Pre-weaning	Yes
NL1	Netherlands	Meningitis	Post-weaning	Yes
NL2	Netherlands	None	None	None
US1	USA	None	None	Yes

Table 2. Antimicrobial administration in farms.

Class	Type used in study piglets:
aminoglycoside	Neomycin (ES3), Gentamicin (ES4, US1)
tetracycline	Tetracycline (DE1, DE3)
beta lactam	Amoxicillin (DE3, DE6, ES2, ES3, ES4, US1)
fluoroquinolone	Marbofloxacin (ES2)

Fam description:

DE1 (Germany)

Regular commercial farm. Intermittent *S. suis* problems. Tetracycline treatment since day 1 for piglet 1-4 but not 5-8. Farm size: Sows: 800, Nursery: 2000.

DE2 (Germany)

Regular commercial farm. Very high health status, and piglets had no problems with any disease in the period around sampling. Prestarter feed provided in both dry and liquid form and open water source provided. The piglet did not have their teeth ground or cut. All piglets were treated with amoxicillin the first day after birth as is typical in German farms. Farm size: Sows: 500, Nursery: 1200.

DE3 (Germany)

Regular commercial farm with some PRRSV problems (no vaccination) and intermittent *S. suis* problems. Low health farm with high antimicrobial usage for months/years. Amoxicillin and Tetracycline treatment for 5 days before sampling of piglets 1-5 but not piglet 6-7. Farm size: Sows: 600, Nursery: 2800.

DE4 (Germany)

Ecological farm utilizing vaccines. The piglets have outside access and straw bedding. The farm has reduced biosecurity, not only due to the outside access and lower level of cleanliness but also due to a lack of separation of airflow between age groups, which may facilitate transfer of pathogens. In addition to *S. suis*, the piglets have a high burden of parasites as well as rotavirus problems. No antimicrobial treatment of the sampled piglets. Farm size: Sows: 300, Nursery: 1200.

DE5 (Germany)

Regular commercial farm. Intermittent *S. suis* problems. No antimicrobial treatment of the sampled piglets. Farm size: Sows: 1200, Nursery: 5000.

DE6 (Germany)

Regular commercial farm. This farm has a history of severe *S. suis* problems. PRRSV and PCV2 (outbreak ongoing at sampling, may relate to failure in vaccination), and other pathogens also cause problems at this farm. Only 1 pig treated with antimicrobials (Amoxicillin) in the days prior to sampling. Farm size: Sows: 100, Nursery: 2000.

ES1 (Spain) - sample prefix QT

Regular commercial farm. Production farm receiving sows from ES2. No history of *S. suis* problems, although this is not a high health status farm in general. No antimicrobials used on sampled piglets. This farm was visited two times, with microbiota sampling of the same piglets at day 14 (1 week before weaning) and day 51 (3 weeks post-weaning). Weaning at day 21.

ES2 (Spain) - sample prefix RC

Regular commercial breeding farm. This farm delivers dams to farm ES1. This farm has a history of severe *S. suis* problems. This farm was followed from 1 week before weaning to 3 weeks post-weaning, with 1 microbiota sampling per week for 5 weeks. Weaning day 26-29. Sampling at day 18-21 (week -1), day 24-27 (week 0), day 32-35 (week +1), day 39-42 (week +2), and day 47-50 (week +3). All piglets were

given amoxicillin in water at day 39, 12 days after weaning and 1 day before sampling point week 2 after weaning. Piglets in some nursery pens were treated with intramuscular marbofloxacin injections, see metadata.

ES3 (Spain) - sample prefix BN
Regular commercial farm. We sampled pre-weaning *S. suis* cases. This farm has a history of severe *S. suis* problems. All piglets were given prestarter feed with amoxicillin and neomycin.

ES4 (Spain) - sample prefix JT
Regular commercial farm. This farm has a history of severe *S. suis* problems. All piglets were given amoxicillin + gentamicin injections 2 days before sampling.

NL1 (Netherlands)
Research farm, Schothorst Feed Research B.V., Lelystad, Netherlands. High health status and low antimicrobial usage. Intermittent *S. suis* problems. No antimicrobials were given to the sampled piglets.

FST (Netherlands)
Piglets free roaming in a forest, with no access to indoor housing. Breed: Tamworth. No vaccination or antimicrobial treatment. The piglets forage for food, but this is also supplemented with some dry feed, fruit, and vegetables. Piglets were weaned and living in a different section than the sows at the time of sampling but spent considerably longer time with the sow than in regular farms (until naturally weaned). The outdoor conditions, rooting in soil, varied diet, gradual weaning, and rare breed may all have contributed to the diverged tonsillar microbiota.

US1 (USA)
Regular commercial farm. A farrow-to-finish farm located in the Midwest USA. Overall high health status. Intermittent *S. suis* problems, usually occurring late nursery or early finisher stage. All piglets were treated with gentamicin (day 26) and amoxicillin (day 45). The piglets were weaned at day 26 and sampled at day 55/62.

Supplementary Table 1. Case-control associations for ASVs

Table S1. Case-control associations for ASVs.

ASV	Case	Control	Ratio	Mean	wilcox_unpaired	wilcox_unpaired_fdr	Sequence	Kingdom	Phylum	Class	Order	Family	Genus	Species	Binomial
ASV00029_Prevotella_Unclassified	1,767	0,688	2,570	1,227	0,001	0,071	TGAGGAATATTGGTCAATGGG	Bacteria	Bacteroidota	Bacteroidia	Bacteroidales	Prevotellaceae	Prevotella	Unclassified	Prevotella_Unclassified
ASV00011_Actinobacillus_minor	0,216	0,637	0,340	0,426	0,002	0,071	TGGGGAATATTGCACAATGGG	Bacteria	Proteobacteria	Gammaproteobacteria	Pasteurellales	Pasteurellaceae	Actinobacillus	minor	Actinobacillus_minor
ASV00021_Fusobacterium_gastrosuis	3,600	1,530	2,353	2,565	0,002	0,071	TGGGGAATATTGGACAATGGA	Bacteria	Fusobacteriota	Fusobacteriia	Fusobacteriales	Fusobacteriaceae	Fusobacterium	gastrosuis	Fusobacterium_gastrosuis
ASV00003_Alloprevotella_Unclassified	3,677	2,194	1,676	2,935	0,002	0,071	TGAGGAATATTGGTCAATGGG	Bacteria	Bacteroidota	Bacteroidia	Bacteroidales	Prevotellaceae	Alloprevotella	Unclassified	Alloprevotella_Unclassified
ASV00081_Terrisporobacter_mayombei	0,214	0,396	0,540	0,305	0,002	0,071	TGGGGAATATTGCACAATGGG	Bacteria	Firmicutes	Clostridia	Peptostreptococcales-Tissierellales	Peptostreptococcaceae	Terrisporobacter	mayombei	Terrisporobacter_mayombei
ASV00137_Bergeyella_porcorum	0,319	0,160	1,990	0,239	0,005	0,149	TGAGGAATATTGGACAATGGG	Bacteria	Bacteroidota	Bacteroidia	Flavobacteriales	Weeksellaceae	Bergeyella	porcorum	Bergeyella_porcorum
ASV00048_Clostridium sensu stricto 1_disporicum	0,381	0,740	0,515	0,561	0,008	0,163	TGGGGAATATTGCACAATGGG	Bacteria	Firmicutes	Clostridia	Clostridiales	Clostridiaceae	Clostridium sensu stricto 1	disporicum	Clostridium sensu stricto 1_disporicum
ASV00132_Agathobacter_Unclassified	0,089	0,204	0,437	0,146	0,008	0,163	TGGGGAATATTGCACAATGGG	Bacteria	Firmicutes	Clostridia	Lachnospirales	Lachnospiraceae	Agathobacter	Unclassified	Agathobacter_Unclassified
ASV00122_Streptococcus_suis	0,200	0,268	0,748	0,234	0,011	0,198	TAGGGAATCTTCGGCAATGGG	Bacteria	Firmicutes	Bacilli	Lactobacillales	Streptococcaceae	Streptococcus	suis	Streptococcus_suis
ASV00036_Rothia_nasimurium	0,478	0,909	0,526	0,693	0,014	0,213	TGGGGAATATTGCACAATGGG	Bacteria	Actinobacteriota	Actinobacteria	Micrococcales	Micrococcaceae	Rothia	nasimurium	Rothia_nasimurium
ASV00001_Moraxella_porci/pluranimalium	3,710	8,145	0,456	5,928	0,014	0,213	TGGGGAATATTGGACAATGGG	Bacteria	Proteobacteria	Gammaproteobacteria	Pseudomonadales	Moraxellaceae	Moraxella	porci/pluranimalium	Moraxella_porci/pluranimalium
ASV00117_Prevotella_copri	0,148	0,291	0,508	0,219	0,016	0,217	TGAGGAATATTGGTCAATGGG	Bacteria	Bacteroidota	Bacteroidia	Bacteroidales	Prevotellaceae	Prevotella	copri	Prevotella_copri
ASV00006_Streptococcus_suis	1,986	2,617	0,759	2,301	0,017	0,217	TAGGGAATCTTCGGCAATGGG	Bacteria	Firmicutes	Bacilli	Lactobacillales	Streptococcaceae	Streptococcus	suis	Streptococcus_suis
ASV00104_Lactobacillus_johnsonii/taiwanensis/prophage/hominis	0,213	0,303	0,705	0,258	0,021	0,241	TAGGGAATCTTCCACAATGGAC	Bacteria	Firmicutes	Bacilli	Lactobacillales	Lactobacillaceae	Lactobacillus	johnsonii/taiwanensis/prophage/hominis	Lactobacillus_johnsonii/taiwanensis/prophage/hominis
ASV00018_Streptococcus_suis	1,600	2,053	0,779	1,826	0,022	0,241	TAGGGAATCTTCGGCAATGGG	Bacteria	Firmicutes	Bacilli	Lactobacillales	Streptococcaceae	Streptococcus	suis	Streptococcus_suis
ASV00034_Actinobacillus_minor	0,130	0,390	0,334	0,260	0,030	0,310	TGGGGAATATTGCACAATGGG	Bacteria	Proteobacteria	Gammaproteobacteria	Pasteurellales	Pasteurellaceae	Actinobacillus	minor	Actinobacillus_minor
ASV00174_Actinobacillus_A. indolicus/H. parasuis	0,263	0,158	1,659	0,211	0,040	0,376	TGGGGAATATTGCACAATGGG	Bacteria	Proteobacteria	Gammaproteobacteria	Pasteurellales	Pasteurellaceae	Actinobacillus	A. indolicus/H. parasuis	Actinobacillus_A. indolicus/H. parasuis
ASV00025_Actinobacillus_minor	0,067	0,162	0,414	0,114	0,041	0,376	TGGGGAATATTGCACAATGGG	Bacteria	Proteobacteria	Gammaproteobacteria	Pasteurellales	Pasteurellaceae	Actinobacillus	minor	Actinobacillus_minor
ASV00195_Moraxella_lacunata	0,162	0,165	0,981	0,164	0,049	0,424	TGGGGAATATTGGACAATGGG	Bacteria	Proteobacteria	Gammaproteobacteria	Pseudomonadales	Moraxellaceae	Moraxella	lacunata	Moraxella_lacunata
ASV00052_Lactobacillus_amylovorus/acidophilus/ultunensis	0,768	0,656	1,171	0,712	0,053	0,437	TAGGGAATCTTCCACAATGGAC	Bacteria	Firmicutes	Bacilli	Lactobacillales	Lactobacillaceae	Lactobacillus	amylovorus/acidophilus/ultunensis	Lactobacillus_amylovorus/acidophilus/ultunensis
ASV00149_Actinobacillus_porcinus	0,372	0,080	4,672	0,226	0,057	0,444	TGGGGAATATTGCACAATGGG	Bacteria	Proteobacteria	Gammaproteobacteria	Pasteurellales	Pasteurellaceae	Actinobacillus	porcinus	Actinobacillus_porcinus
ASV00125_Dielma_Unclassified	0,429	0,147	2,915	0,288	0,064	0,483	TAGGGAATTTTCCACAATGGG	Bacteria	Firmicutes	Bacilli	Erysipelotrichales	Erysipelotrichaceae	Dielma	Unclassified	Dielma_Unclassified
ASV00017_Streptococcus_suis	0,599	0,985	0,608	0,792	0,077	0,513	TAGGGAATCTTCGGCAATGGG	Bacteria	Firmicutes	Bacilli	Lactobacillales	Streptococcaceae	Streptococcus	suis	Streptococcus_suis
ASV00127_Blautia_obeum/wexlerae	0,149	0,173	0,860	0,161	0,077	0,513	TGGGGAATATTGCACAATGGG	Bacteria	Firmicutes	Clostridia	Lachnospirales	Lachnospiraceae	Blautia	obeum/wexlerae	Blautia_obeum/wexlerae
ASV00046_Conchiformibius_Unclassified	0,408	0,485	0,841	0,447	0,079	0,513	TGGGGAATTTTGGACAATGGG	Bacteria	Proteobacteria	Gammaproteobacteria	Burkholderiales	Neisseriaceae	Conchiformibius	Unclassified	Conchiformibius_Unclassified
ASV00136_Alloprevotella_Unclassified	0,392	0,118	3,316	0,255	0,081	0,513	TGAGGAATATTGGTCAATGGA	Bacteria	Bacteroidota	Bacteroidia	Bacteroidales	Prevotellaceae	Alloprevotella	Unclassified	Alloprevotella_Unclassified
ASV00236_Prevotella_Unclassified	0,320	0,091	3,526	0,205	0,086	0,523	TGAGGAATATTGGTCAATGGA	Bacteria	Bacteroidota	Bacteroidia	Bacteroidales	Prevotellaceae	Prevotella	Unclassified	Prevotella_Unclassified
ASV00205_Actinobacillus_minor	0,239	0,166	1,437	0,203	0,094	0,537	TGGGGAATATTGCACAATGGG	Bacteria	Proteobacteria	Gammaproteobacteria	Pasteurellales	Pasteurellaceae	Actinobacillus	minor	Actinobacillus_minor
ASV00165_Johnsonella_Unclassified	0,243	0,163	1,490	0,203	0,094	0,537	TGGGGAATATTGGACAATGGG	Bacteria	Firmicutes	Clostridia	Lachnospirales	Lachnospiraceae	Johnsonella	Unclassified	Johnsonella_Unclassified
ASV00102_Actinobacillus_indolicus/minor	0,153	0,427	0,358	0,290	0,113	0,583	TGGGGAATATTGCACAATGGG	Bacteria	Proteobacteria	Gammaproteobacteria	Pasteurellales	Pasteurellaceae	Actinobacillus	indolicus/minor	Actinobacillus_indolicus/minor
ASV00016_Actinobacillus_indolicus/minor	1,258	1,634	0,770	1,446	0,120	0,583	TGGGGAATATTGCACAATGGG	Bacteria	Proteobacteria	Gammaproteobacteria	Pasteurellales	Pasteurellaceae	Actinobacillus	indolicus/minor	Actinobacillus_indolicus/minor
ASV00134_Porphyromonas_Unclassified	0,270	0,145	1,865	0,208	0,123	0,583	TGAGGAATATTGGTCAATGGG	Bacteria	Bacteroidota	Bacteroidia	Bacteroidales	Porphyromonadaceae	Porphyromonas	Unclassified	Porphyromonas_Unclassified
ASV00148_Lactobacillus_reuteri/fermentum/vaginalis/gasseri	0,153	0,167	0,919	0,160	0,131	0,583	TAGGGAATCTTCCACAATGGG	Bacteria	Firmicutes	Bacilli	Lactobacillales	Lactobacillaceae	Lactobacillus	reuteri/fermentum/vaginalis/gasseri	Lactobacillus_reuteri/fermentum/vaginalis/gasseri
ASV00038_Fusobacterium_necrophorum	1,241	0,755	1,644	0,998	0,132	0,583	TGGGGAATATTGGACAATGGA	Bacteria	Fusobacteriota	Fusobacteriia	Fusobacteriales	Fusobacteriaceae	Fusobacterium	necrophorum	Fusobacterium_necrophorum
ASV00073_Unclassified_Unclassified	0,229	0,532	0,431	0,381	0,144	0,583	TGGGGAATTTTGGACAATGGG	Bacteria	Proteobacteria	Gammaproteobacteria	Burkholderiales	Neisseriaceae	Unclassified	Unclassified	Unclassified_Unclassified
ASV00098_Lactobacillus_salivarius/phage/reuteri	0,147	0,166	0,883	0,157	0,147	0,583	TAGGGAATCTTCCACAATGGAC	Bacteria	Firmicutes	Bacilli	Lactobacillales	Lactobacillaceae	Lactobacillus	salivarius/phage/reuteri	Lactobacillus_salivarius/phage/reuteri
ASV00007_Actinobacillus_porcinus	1,543	1,806	0,854	1,675	0,148	0,583	TGGGGAATATTGCACAATGGG	Bacteria	Proteobacteria	Gammaproteobacteria	Pasteurellales	Pasteurellaceae	Actinobacillus	porcinus	Actinobacillus_porcinus
ASV00193_Bacteroides_Unclassified	0,172	0,184	0,932	0,178	0,150	0,583	TGAGGAATATTGGTCAATGGG	Bacteria	Bacteroidota	Bacteroidia	Bacteroidales	Bacteroidaceae	Bacteroides	Unclassified	Bacteroides_Unclassified
ASV00092_Unclassified_Unclassified	0,430	0,278	1,550	0,354	0,152	0,583	TGGGGAATATTGGGCAATGGG	Bacteria	Firmicutes	Clostridia	Hungateiclostridiaceae	Mageibacillus	Unclassified	Unclassified	Unclassified_Unclassified
ASV00085_Porphyromonas_Unclassified	0,683	0,569	1,200	0,626	0,154	0,583	TGAGGAATATTGGTCAATGGG	Bacteria	Bacteroidota	Bacteroidia	Bacteroidales	Porphyromonadaceae	Porphyromonas	Unclassified	Porphyromonas_Unclassified
ASV00040_Bergeyella_zoohelcum	0,820	0,608	1,348	0,714	0,156	0,583	TGAGGAATATTGGACAATGGG	Bacteria	Bacteroidota	Bacteroidia	Flavobacteriales	Weeksellaceae	Bergeyella	zoohelcum	Bergeyella_zoohelcum
ASV00113_Actinobacillus_minor/indolicus	0,258	0,365	0,706	0,311	0,157	0,583	TGGGGAATATTGCACAATGGG	Bacteria	Proteobacteria	Gammaproteobacteria	Pasteurellales	Pasteurellaceae	Actinobacillus	minor/indolicus	Actinobacillus_minor/indolicus
ASV00109_Streptococcus_suis	0,194	0,437	0,444	0,315	0,163	0,583	TAGGGAATCTTCGGCAATGGG	Bacteria	Firmicutes	Bacilli	Lactobacillales	Streptococcaceae	Streptococcus	suis	Streptococcus_suis
ASV00158_Dysgonomonas_Unclassified	0,170	0,139	1,225	0,154	0,164	0,583	TGAGGAATATTGGTCAATGGG	Bacteria	Bacteroidota	Bacteroidia	Bacteroidales	Dysgonomonadaceae	Dysgonomonas	Unclassified	Dysgonomonas_Unclassified
ASV00199_Porphyromonas_Unclassified	0,298	0,135	2,208	0,216	0,165	0,583	TGAGGAATATTGGTCAATGGG	Bacteria	Bacteroidota	Bacteroidia	Bacteroidales	Porphyromonadaceae	Porphyromonas	Unclassified	Porphyromonas_Unclassified
ASV00227_Conchiformibius_Unclassified	0,227	0,099	2,290	0,163	0,167	0,583	TGGGGAATTTTGGACAATGGG	Bacteria	Proteobacteria	Gammaproteobacteria	Burkholderiales	Neisseriaceae	Conchiformibius	Unclassified	Conchiformibius_Unclassified
ASV00196_Alloprevotella_Unclassified	0,281	0,223	1,260	0,252	0,167	0,583	TGAGGAATATTGGTCAATGGG	Bacteria	Bacteroidota	Bacteroidia	Bacteroidales	Prevotellaceae	Alloprevotella	Unclassified	Alloprevotella_Unclassified
ASV00128_Fusobacterium_nucleatum	0,161	0,207	0,778	0,184	0,170	0,583	TGGGGAATATTGGACAATGGA	Bacteria	Fusobacteriota	Fusobacteriia	Fusobacteriales	Fusobacteriaceae	Fusobacterium	nucleatum	Fusobacterium_nucleatum
ASV00055_Actinobacillus_minor	1,162	1,060	1,096	1,111	0,175	0,590	TGGGGAATATTGCACAATGGG	Bacteria	Proteobacteria	Gammaproteobacteria	Pasteurellales	Pasteurellaceae	Actinobacillus	minor	Actinobacillus_minor
ASV00184_Campylobacter_mucosalis	0,138	0,136	1,014	0,137	0,192	0,616	TAGGGAATATTGCTCAATGGG	Bacteria	Campilobacterota	Campylobacteria	Campylobacterales	Campylobacteraceae	Campylobacter	mucosalis	Campylobacter_mucosalis
ASV00100_Rothia_endophytica/marina	0,318	0,339	0,936	0,329	0,194	0,616	TGGGGAATATTGCACAATGGG	Bacteria	Actinobacteriota	Actinobacteria	Micrococcales	Micrococcaceae	Rothia	endophytica/marina	Rothia_endophytica/marina
ASV00202_Actinobacillus_minor/indolicus	0,121	0,171	0,706	0,146	0,197	0,616	TGGGGAATATTGCACAATGGG	Bacteria	Proteobacteria	Gammaproteobacteria	Pasteurellales	Pasteurellaceae	Actinobacillus	minor/indolicus	Actinobacillus_minor/indolicus
ASV00107_Fusobacterium_gastrosuis	0,534	0,325	1,644	0,429	0,198	0,616	TGGGGAATATTGGACAATGGA	Bacteria	Fusobacteriota	Fusobacteriia	Fusobacteriales	Fusobacteriaceae	Fusobacterium	gastrosuis	Fusobacterium_gastrosuis
ASV00030_Actinobacillus_indolicus	0,330	0,478	0,690	0,404	0,207	0,620	TGGGGAATATTGCACAATGGG	Bacteria	Proteobacteria	Gammaproteobacteria	Pasteurellales	Pasteurellaceae	Actinobacillus	indolicus	Actinobacillus_indolicus
ASV00101_Moraxella_porci/pluranimalium	0,263	0,265	0,991	0,264	0,216	0,620	TGGGGAATATTGGACAATGGG	Bacteria	Proteobacteria	Gammaproteobacteria	Pseudomonadales	Moraxellaceae	Moraxella	porci/pluranimalium	Moraxella_porci/pluranimalium
ASV00061_Actinobacillus_minor/porcitonsillarum	0,139	0,212	0,655	0,175	0,217	0,620	TGGGGAATATTGCACAATGGG	Bacteria	Proteobacteria	Gammaproteobacteria	Pasteurellales	Pasteurellaceae	Actinobacillus	minor/porcitonsillarum	Actinobacillus_minor/porcitonsillarum
ASV00014_Actinobacillus_indolicus	0,477	0,790	0,604	0,634	0,218	0,620	TGGGGAATATTGCACAATGGG	Bacteria	Proteobacteria	Gammaproteobacteria	Pasteurellales	Pasteurellaceae	Actinobacillus	indolicus	Actinobacillus_indolicus
ASV00076_Unclassified_Unclassified	0,254	0,349	0,728	0,301	0,218	0,620	TAGGGAATCTTCGGCAATGGA	Bacteria	Firmicutes	Bacilli	Lactobacillales	Unclassified	Unclassified	Unclassified	Unclassified_Unclassified
ASV00151_Peptostreptococcus_stomatis	0,283	0,161	1,757	0,222	0,230	0,638	TGGGGAATATTGCACAATGGG	Bacteria	Firmicutes	Clostridia	Peptostreptococcales-Tissierellales	Peptostreptococcaceae	Peptostreptococcus	stomatis	Peptostreptococcus_stomatis
ASV00064_Actinobacillus_A. indolicus/H. parasuis	0,569	0,510	1,115	0,539	0,239	0,638	TGGGGAATATTGCACAATGGG	Bacteria	Proteobacteria	Gammaproteobacteria	Pasteurellales	Pasteurellaceae	Actinobacillus	A. indolicus/H. parasuis	Actinobacillus_A. indolicus/H. parasuis
ASV00103_Actinobacillus_indolicus/minor/porcitonsillarum	0,327	0,409	0,798	0,368	0,240	0,638	TGGGGAATATTGCACAATGGG	Bacteria	Proteobacteria	Gammaproteobacteria	Pasteurellales	Pasteurellaceae	Actinobacillus	indolicus/minor/porcitonsillarum	Actinobacillus_indolicus/minor/porcitonsillarum
ASV00013_Moraxella_porci	3,068	2,850	1,076	2,959	0,240	0,638	TGGGGAATATTGGACAATGGG	Bacteria	Proteobacteria	Gammaproteobacteria	Pseudomonadales	Moraxellaceae	Moraxella	porci	Moraxella_porci
ASV00057_Escherichia-Shigella_fergusonii/coli/sonnei/flexneri/dysenter	0,346	0,431	0,803	0,388	0,252	0,658	TGGGGAATATTGCACAATGGG	Bacteria	Proteobacteria	Gammaproteobacteria	Enterobacterales	Enterobacteriaceae	Escherichia-Shigella	fergusonii/coli/sonnei/flexneri/dysenteriae/albertii	Escherichia-Shigella_fergusonii/coli/sonnei/flexneri/dysenteriae/albertii
ASV00171_Acinetobacter_schindleri/johnsonii	0,475	0,000	1059,464	0,238	0,260	0,658	TGGGGAATATTGGACAATGGG	Bacteria	Proteobacteria	Gammaproteobacteria	Pseudomonadales	Moraxellaceae	Acinetobacter	schindleri/johnsonii	Acinetobacter_schindleri/johnsonii
ASV00206_Johnsonella_Unclassified	0,184	0,135	1,362	0,160	0,262	0,658	TGGGGAATATTGGACAATGGG	Bacteria	Firmicutes	Clostridia	Lachnospirales	Lachnospiraceae	Johnsonella	Unclassified	Johnsonella_Unclassified
ASV00019_Streptococcus_suis	1,018	1,344	0,757	1,181	0,273	0,658	TAGGGAATCTTCGGCAATGGG	Bacteria	Firmicutes	Bacilli	Lactobacillales	Streptococcaceae	Streptococcus	suis	Streptococcus_suis
ASV00027_Actinobacillus_A. indolicus/H. parasuis	1,036	1,274	0,813	1,155	0,279	0,658	TGGGGAATATTGCACAATGGG	Bacteria	Proteobacteria	Gammaproteobacteria	Pasteurellales	Pasteurellaceae	Actinobacillus	A. indolicus/H. parasuis	Actinobacillus_A. indolicus/H. parasuis
ASV00138_Porphyromonas_Unclassified	0,139	0,206	0,675	0,173	0,279	0,658	TGAGGAATATTGGTCAATGGG	Bacteria	Bacteroidota	Bacteroidia	Bacteroidales	Porphyromonadaceae	Porphyromonas	Unclassified	Porphyromonas_Unclassified
ASV00039_Alloprevotella_Unclassified	0,939	0,755	1,244	0,847	0,280	0,658	TGAGGAATATTGGTCAATGGG	Bacteria	Bacteroidota	Bacteroidia	Bacteroidales	Prevotellaceae	Alloprevotella	Unclassified	Alloprevotella_Unclassified
ASV00032_Actinobacillus_porcinus	0,634	0,651	0,974	0,643	0,282	0,658	TGGGGAATATTGCACAATGGG	Bacteria	Proteobacteria	Gammaproteobacteria	Pasteurellales	Pasteurellaceae	Actinobacillus	porcinus	Actinobacillus_porcinus
ASV00080_Actinobacillus_porcinus	0,561	0,427	1,316	0,494	0,283	0,658	TGGGGAATATTGCACAATGGG	Bacteria	Proteobacteria	Gammaproteobacteria	Pasteurellales	Pasteurellaceae	Actinobacillus	porcinus	Actinobacillus_porcinus
ASV00223_Unclassified_minor/indolicus	0,067	0,218	0,306	0,142	0,306	0,702	TGGGGAATATTGCACAATGGG	Bacteria	Proteobacteria	Gammaproteobacteria	Pasteurellales	Pasteurellaceae	Unclassified	minor/indolicus	Unclassified_minor/indolicus
ASV00160_Actinobacillus_Unclassified	0,325	0,108	2,994	0,216	0,313	0,708	TGGGGAATATTGCGCAATGGG	Bacteria	Proteobacteria	Gammaproteobacteria	Pasteurellales	Pasteurellaceae	Actinobacillus	Unclassified	Actinobacillus_Unclassified
ASV00091_Unclassified_Unclassified	0,355	0,290	1,223	0,323	0,328	0,730	TGGGGAATATTGGACAATGGG	Bacteria	Firmicutes	Clostridia	Lachnospirales	Lachnospiraceae	Unclassified	Unclassified	Unclassified_Unclassified
ASV00026_Actinobacillus_indolicus	0,442	0,547	0,807	0,495	0,332	0,730	TGGGGAATATTGCACAATGGG	Bacteria	Proteobacteria	Gammaproteobacteria	Pasteurellales	Pasteurellaceae	Actinobacillus	indolicus	Actinobacillus_indolicus
ASV00049_Acinetobacter_Unclassified	0,667	0,657	1,016	0,662	0,355	0,732	TGGGGAATATTGGACAATGGG	Bacteria	Proteobacteria	Gammaproteobacteria	Pseudomonadales	Moraxellaceae	Acinetobacter	Unclassified	Acinetobacter_Unclassified
ASV00095_Prevotella_Unclassified	0,120	0,199	0,602	0,160	0,360	0,732	TGAGGAATATTGGTCAATGGG	Bacteria	Bacteroidota	Bacteroidia	Bacteroidales	Prevotellaceae	Prevotella	Unclassified	Prevotella_Unclassified
ASV00169_Unclassified_aerogenes	0,171	0,176	0,971	0,173	0,361	0,732	TGGGGAATATTGCGCAATGGG	Bacteria	Proteobacteria	Gammaproteobacteria	Pasteurellales	Pasteurellaceae	Unclassified	aerogenes	Unclassified_aerogenes
ASV00063_Unclassified_Unclassified	0,824	0,547	1,506	0,686	0,363	0,732	TGGGGAATATTGGACAATGGG	Bacteria	Proteobacteria	Gammaproteobacteria	Pseudomonadales	Moraxellaceae	Unclassified	Unclassified	Unclassified_Unclassified
ASV00033_Alloprevotella_Unclassified	1,103	0,812	1,358	0,958	0,367	0,732	TGAGGAATATTGGTCAATGGG	Bacteria	Bacteroidota	Bacteroidia	Bacteroidales	Prevotellaceae	Alloprevotella	Unclassified	Alloprevotella_Unclassified
ASV00197_Bordetella_bronchiseptica/parapertussis	0,044	0,325	0,136	0,185	0,367	0,732	TGGGGAATTTTGGACAATGGG	Bacteria	Proteobacteria	Gammaproteobacteria	Burkholderiales	Alcaligenaceae	Bordetella	bronchiseptica/parapertussis	Bordetella_bronchiseptica/parapertussis
ASV00024_Moraxella_boevrei/sonnei	0,245	0,347	0,706	0,296	0,369	0,732	TGGGGAATATTGGACAATGGG	Bacteria	Proteobacteria	Gammaproteobacteria	Pseudomonadales	Moraxellaceae	Moraxella	boevrei/sonnei	Moraxella_boevrei/sonnei
ASV00047_Actinobacillus_porcinus	0,141	0,167	0,842	0,154	0,370	0,732	TGGGGAATATTGCACAATGGG	Bacteria	Proteobacteria	Gammaproteobacteria	Pasteurellales	Pasteurellaceae	Actinobacillus	porcinus	Actinobacillus_porcinus
ASV00156_Alloprevotella_Unclassified	0,182	0,165	1,108	0,173	0,373	0,732	TGAGGAATATTGGTCAATGGG	Bacteria	Bacteroidota	Bacteroidia	Bacteroidales	Prevotellaceae	Alloprevotella	Unclassified	Alloprevotella_Unclassified
ASV00082_Actinobacillus_indolicus	0,518	0,471	1,100	0,494	0,386	0,735	TGGGGAATATTGCACAATGGG	Bacteria	Proteobacteria	Gammaproteobacteria	Pasteurellales	Pasteurellaceae	Actinobacillus	indolicus	Actinobacillus_indolicus
ASV00123_Conchiformibius_Unclassified	0,497	0,301	1,651	0,399	0,388	0,735	TGGGGAATTTTGGACAATGGG	Bacteria	Proteobacteria	Gammaproteobacteria	Burkholderiales	Neisseriaceae	Conchiformibius	Unclassified	Conchiformibius_Unclassified
ASV00159_Neisseria_dentiae	0,259	0,147	1,762	0,203	0,389	0,735	TGGGGAATTTTGGACAATGGG	Bacteria	Proteobacteria	Gammaproteobacteria	Burkholderiales	Neisseriaceae	Neisseria	dentiae	Neisseria_dentiae
ASV00066_Streptobacillus_Unclassified	0,524	0,486	1,078	0,505	0,393	0,735	TGGGGAATATTGGACAATGGA	Bacteria	Fusobacteriota	Fusobacteriia	Fusobacteriales	Leptotrichiaceae	Streptobacillus	Unclassified	Streptobacillus_Unclassified
ASV00175_Streptococcus_salivarius/vestibularis/cristatus/albilineans/p	0,067	0,192	0,350	0,130	0,396	0,735	TAGGGAATCTTCGGCAATGGG	Bacteria	Firmicutes	Bacilli	Lactobacillales	Streptococcaceae	Streptococcus	salivarius/vestibularis/cristatus/albilineans/pneumoniae/equinus	Streptococcus_salivarius/vestibularis/cristatus/albilineans/pneumoniae/equinus
ASV00031_Actinobacillus_minor/porcitonsillarum	0,832	0,900	0,925	0,866	0,406	0,743	TGGGGAATATTGCACAATGGG	Bacteria	Proteobacteria	Gammaproteobacteria	Pasteurellales	Pasteurellaceae	Actinobacillus	minor/porcitonsillarum	Actinobacillus_minor/porcitonsillarum
ASV00010_Actinobacillus_indolicus/minor/porcitonsillarum	2,447	2,149	1,139	2,298	0,410	0,743	TGGGGAATATTGCACAATGGG	Bacteria	Proteobacteria	Gammaproteobacteria	Pasteurellales	Pasteurellaceae	Actinobacillus	indolicus/minor/porcitonsillarum	Actinobacillus_indolicus/minor/porcitonsillarum
ASV00022_Actinobacillus_minor	2,432	2,306	1,054	2,369	0,415	0,744	TGGGGAATATTGCACAATGGG	Bacteria	Proteobacteria	Gammaproteobacteria	Pasteurellales	Pasteurellaceae	Actinobacillus	minor	Actinobacillus_minor
ASV00042_Acinetobacter_Unclassified	0,261	0,295	0,886	0,278	0,424	0,749	TGGGGAATATTGGACAATGGG	Bacteria	Proteobacteria	Gammaproteobacteria	Pseudomonadales	Moraxellaceae	Acinetobacter	Unclassified	Acinetobacter_Unclassified
ASV00071_Actinobacillus_A. indolicus/H. parasuis	0,266	0,286	0,930	0,276	0,427	0,749	TGGGGAATATTGCACAATGGG	Bacteria	Proteobacteria	Gammaproteobacteria	Pasteurellales	Pasteurellaceae	Actinobacillus	A. indolicus/H. parasuis	Actinobacillus_A. indolicus/H. parasuis
ASV00190_Streptococcus_Unclassified	0,200	0,147	1,360	0,174	0,433	0,752	TAGGGAATCTTCGGCAATGGG	Bacteria	Firmicutes	Bacilli	Lactobacillales	Streptococcaceae	Streptococcus	Unclassified	Streptococcus_Unclassified
ASV00133_Veillonella_parvula	0,189	0,175	1,080	0,182	0,441	0,758	TGGGGAATCTTCCGCAATGGA	Bacteria	Firmicutes	Negativicutes	Veillonellales-Selenomonadales	Veillonellaceae	Veillonella	parvula	Veillonella_parvula
ASV00096_Streptococcus_henryi/orisratti/suis/porcorum	0,547	0,366	1,494	0,457	0,461	0,784	TAGGGAATCTTCGGCAATGGG	Bacteria	Firmicutes	Bacilli	Lactobacillales	Streptococcaceae	Streptococcus	henryi/orisratti/suis/porcorum	Streptococcus_henryi/orisratti/suis/porcorum
ASV00004_Streptococcus_porcorum	1,512	2,010	0,752	1,761	0,476	0,786	TAGGGAATCTTCGGCAATGGG	Bacteria	Firmicutes	Bacilli	Lactobacillales	Streptococcaceae	Streptococcus	porcorum	Streptococcus_porcorum
ASV00075_Actinobacillus_porcinus	0,261	0,216	1,208	0,238	0,476	0,786	TGGGGAATATTGCACAATGGG	Bacteria	Proteobacteria	Gammaproteobacteria	Pasteurellales	Pasteurellaceae	Actinobacillus	porcinus	Actinobacillus_porcinus
ASV00180_Dysgonomonas_Unclassified	0,095	0,137	0,697	0,116	0,477	0,786	TGAGGAATATTGGTCAATGGG	Bacteria	Bacteroidota	Bacteroidia	Bacteroidales	Dysgonomonadaceae	Dysgonomonas	Unclassified	Dysgonomonas_Unclassified
ASV00094_Alloprevotella_Unclassified	0,435	0,446	0,976	0,441	0,481	0,786	TGAGGAATATTGGTCAATGGG	Bacteria	Bacteroidota	Bacteroidia	Bacteroidales	Prevotellaceae	Alloprevotella	Unclassified	Alloprevotella_Unclassified
ASV00126_Streptococcus_plurextorum	0,196	0,138	1,421	0,167	0,493	0,798	TAGGGAATCTTCGGCAATGGG	Bacteria	Firmicutes	Bacilli	Lactobacillales	Streptococcaceae	Streptococcus	plurextorum	Streptococcus_plurextorum
ASV00150_Bergeyella_zoohelcum	0,081	0,207	0,392	0,144	0,505	0,805	TGAGGAATATTGGACAATGGG	Bacteria	Bacteroidota	Bacteroidia	Flavobacteriales	Weeksellaceae	Bergeyella	zoohelcum	Bergeyella_zoohelcum
ASV00183_Actinobacillus_indolicus/minor/porcitonsillarum	0,218	0,175	1,245	0,197	0,508	0,805	TGGGGAATATTGCACAATGGG	Bacteria	Proteobacteria	Gammaproteobacteria	Pasteurellales	Pasteurellaceae	Actinobacillus	indolicus/minor/porcitonsillarum	Actinobacillus_indolicus/minor/porcitonsillarum
ASV00185_Johnsonella_Unclassified	0,197	0,173	1,140	0,185	0,533	0,822	TGGGGAATATTGGACAATGGG	Bacteria	Firmicutes	Clostridia	Lachnospirales	Lachnospiraceae	Johnsonella	Unclassified	Johnsonella_Unclassified
ASV00023_Actinobacillus_minor/indolicus	1,302	1,251	1,040	1,277	0,535	0,822	TGGGGAATATTGCACAATGGG	Bacteria	Proteobacteria	Gammaproteobacteria	Pasteurellales	Pasteurellaceae	Actinobacillus	minor/indolicus	Actinobacillus_minor/indolicus
ASV00015_Actinobacillus_minor/porcitonsillarum	2,621	3,124	0,839	2,872	0,539	0,822	TGGGGAATATTGCACAATGGG	Bacteria	Proteobacteria	Gammaproteobacteria	Pasteurellales	Pasteurellaceae	Actinobacillus	minor/porcitonsillarum	Actinobacillus_minor/porcitonsillarum
ASV00028_Acinetobacter_Unclassified	1,588	1,263	1,257	1,425	0,546	0,822	TGGGGAATATTGGACAATGGG	Bacteria	Proteobacteria	Gammaproteobacteria	Pseudomonadales	Moraxellaceae	Acinetobacter	Unclassified	Acinetobacter_Unclassified
ASV00214_Actinobacillus_minor/porcitonsillarum	0,106	0,285	0,373	0,196	0,548	0,822	TGGGGAATATTGCACAATGGG	Bacteria	Proteobacteria	Gammaproteobacteria	Pasteurellales	Pasteurellaceae	Actinobacillus	minor/porcitonsillarum	Actinobacillus_minor/porcitonsillarum
ASV00045_Moraxella_Unclassified	0,192	0,255	0,751	0,223	0,556	0,822	TGGGGAATATTGGACAATGGG	Bacteria	Proteobacteria	Gammaproteobacteria	Pseudomonadales	Moraxellaceae	Moraxella	Unclassified	Moraxella_Unclassified
ASV00056_Porphyromonas_Unclassified	0,751	0,747	1,006	0,749	0,562	0,822	TGAGGAATATTGGTCAATGGG	Bacteria	Bacteroidota	Bacteroidia	Bacteroidales	Porphyromonadaceae	Porphyromonas	Unclassified	Porphyromonas_Unclassified
ASV00020_Porphyromonas_Unclassified	2,519	2,016	1,249	2,268	0,573	0,822	TGAGGAATATTGGTCAATGGG	Bacteria	Bacteroidota	Bacteroidia	Bacteroidales	Porphyromonadaceae	Porphyromonas	Unclassified	Porphyromonas_Unclassified
ASV00043_Actinobacillus_porcinus	0,717	0,549	1,306	0,633	0,574	0,822	TGGGGAATATTGCACAATGGG	Bacteria	Proteobacteria	Gammaproteobacteria	Pasteurellales	Pasteurellaceae	Actinobacillus	porcinus	Actinobacillus_porcinus
ASV00208_Neisseria_shayeganii	0,326	0,103	3,163	0,215	0,577	0,822	TGGGGAATTTTGGACAATGGG	Bacteria	Proteobacteria	Gammaproteobacteria	Burkholderiales	Neisseriaceae	Neisseria	shayeganii	Neisseria_shayeganii
ASV00074_Alysiella_Unclassified	0,466	0,755	0,617	0,611	0,581	0,822	TGGGGAATTTTGGACAATGGG	Bacteria	Proteobacteria	Gammaproteobacteria	Burkholderiales	Neisseriaceae	Alysiella	Unclassified	Alysiella_Unclassified
ASV00153_Actinobacillus_indolicus	0,193	0,154	1,249	0,174	0,590	0,822	TGGGGAATATTGCACAATGGG	Bacteria	Proteobacteria	Gammaproteobacteria	Pasteurellales	Pasteurellaceae	Actinobacillus	indolicus	Actinobacillus_indolicus
ASV00203_Porphyromonas_Unclassified	0,199	0,179	1,111	0,189	0,592	0,822	TGAGGAATATTGGTCAATGGG	Bacteria	Bacteroidota	Bacteroidia	Bacteroidales	Porphyromonadaceae	Porphyromonas	Unclassified	Porphyromonas_Unclassified
ASV00108_Bacteroides_Unclassified	0,523	0,323	1,620	0,423	0,592	0,822	TGAGGAATATTGGTCAATGGG	Bacteria	Bacteroidota	Bacteroidia	Bacteroidales	Bacteroidaceae	Bacteroides	Unclassified	Bacteroides_Unclassified
ASV00041_Actinobacillus_indolicus/minor/porcitonsillarum	0,139	0,245	0,569	0,192	0,593	0,822	TGGGGAATATTGCACAATGGG	Bacteria	Proteobacteria	Gammaproteobacteria	Pasteurellales	Pasteurellaceae	Actinobacillus	indolicus/minor/porcitonsillarum	Actinobacillus_indolicus/minor/porcitonsillarum
ASV00008_Actinobacillus_A. indolicus/H. parasuis	1,016	1,233	0,824	1,125	0,602	0,827	TGGGGAATATTGCACAATGGG	Bacteria	Proteobacteria	Gammaproteobacteria	Pasteurellales	Pasteurellaceae	Actinobacillus	A. indolicus/H. parasuis	Actinobacillus_A. indolicus/H. parasuis
ASV00077_Leptotrichia_Unclassified	0,438	0,439	0,998	0,439	0,607	0,828	TGGGGAATATTGGACAATGGA	Bacteria	Fusobacteriota	Fusobacteriia	Fusobacteriales	Leptotrichiaceae	Leptotrichia	Unclassified	Leptotrichia_Unclassified
ASV00120_Streptococcus_Unclassified	0,342	0,219	1,564	0,281	0,623	0,843	TAGGGAATCTTCGGCAATGGG	Bacteria	Firmicutes	Bacilli	Lactobacillales	Streptococcaceae	Streptococcus	Unclassified	Streptococcus_Unclassified
ASV00143_Unclassified_Unclassified	0,211	0,370	0,571	0,290	0,637	0,854	TGGGGAATTTTGGACAATGGG	Bacteria	Proteobacteria	Gammaproteobacteria	Burkholderiales	Neisseriaceae	Unclassified	Unclassified	Unclassified_Unclassified
ASV00106_Acinetobacter_Unclassified	0,360	0,153	2,346	0,257	0,655	0,872	TGGGGAATATTGGACAATGGG	Bacteria	Proteobacteria	Gammaproteobacteria	Pseudomonadales	Moraxellaceae	Acinetobacter	Unclassified	Acinetobacter_Unclassified
ASV00178_Actinobacillus_minor/indolicus	0,216	0,297	0,726	0,256	0,661	0,873	TGGGGAATATTGCACAATGGG	Bacteria	Proteobacteria	Gammaproteobacteria	Pasteurellales	Pasteurellaceae	Actinobacillus	minor/indolicus	Actinobacillus_minor/indolicus
ASV00161_Actinobacillus_porcinus	0,262	0,080	3,273	0,171	0,667	0,874	TGGGGAATATTGCACAATGGG	Bacteria	Proteobacteria	Gammaproteobacteria	Pasteurellales	Pasteurellaceae	Actinobacillus	porcinus	Actinobacillus_porcinus
ASV00118_Actinobacillus_indolicus	0,383	0,253	1,515	0,318	0,675	0,877	TGGGGAATATTGCACAATGGG	Bacteria	Proteobacteria	Gammaproteobacteria	Pasteurellales	Pasteurellaceae	Actinobacillus	indolicus	Actinobacillus_indolicus
ASV00051_Veillonella_caviae	0,615	0,632	0,973	0,623	0,688	0,882	TGGGGAATCTTCCGCAATGGA	Bacteria	Firmicutes	Negativicutes	Veillonellales-Selenomonadales	Veillonellaceae	Veillonella	caviae	Veillonella_caviae
ASV00250_Actinobacillus_indolicus	0,064	0,229	0,281	0,147	0,691	0,882	TGGGGAATATTGCACAATGGG	Bacteria	Proteobacteria	Gammaproteobacteria	Pasteurellales	Pasteurellaceae	Actinobacillus	indolicus	Actinobacillus_indolicus
ASV00154_Actinobacillus_rossii	0,258	0,294	0,879	0,276	0,695	0,882	TGGGGAATATTGCACAATGGG	Bacteria	Proteobacteria	Gammaproteobacteria	Pasteurellales	Pasteurellaceae	Actinobacillus	rossii	Actinobacillus_rossii
ASV00166_Actinobacillus_minor	0,256	0,308	0,830	0,282	0,701	0,883	TGGGGAATATTGCACAATGGG	Bacteria	Proteobacteria	Gammaproteobacteria	Pasteurellales	Pasteurellaceae	Actinobacillus	minor	Actinobacillus_minor
ASV00130_Actinobacillus_minor	0,173	0,160	1,080	0,167	0,728	0,901	TGGGGAATATTGCACAATGGG	Bacteria	Proteobacteria	Gammaproteobacteria	Pasteurellales	Pasteurellaceae	Actinobacillus	minor	Actinobacillus_minor
ASV00012_Actinobacillus_indolicus	0,474	0,545	0,869	0,510	0,732	0,901	TGGGGAATATTGCACAATGGG	Bacteria	Proteobacteria	Gammaproteobacteria	Pasteurellales	Pasteurellaceae	Actinobacillus	indolicus	Actinobacillus_indolicus
ASV00090_Bacteroides_Unclassified	0,322	0,384	0,840	0,353	0,736	0,901	TGAGGAATATTGGTCAATGGA	Bacteria	Bacteroidota	Bacteroidia	Bacteroidales	Bacteroidaceae	Bacteroides	Unclassified	Bacteroides_Unclassified
ASV00065_Actinobacillus_minor/porcitonsillarum	0,313	0,491	0,637	0,402	0,737	0,901	TGGGGAATATTGCACAATGGG	Bacteria	Proteobacteria	Gammaproteobacteria	Pasteurellales	Pasteurellaceae	Actinobacillus	minor/porcitonsillarum	Actinobacillus_minor/porcitonsillarum
ASV00084_Fusobacterium_gastrosuis	0,512	0,402	1,273	0,457	0,748	0,907	TGGGGAATATTGGACAATGGA	Bacteria	Fusobacteriota	Fusobacteriia	Fusobacteriales	Fusobacteriaceae	Fusobacterium	gastrosuis	Fusobacterium_gastrosuis
ASV00141_Fusobacterium_gastrosuis	0,367	0,224	1,636	0,295	0,756	0,910	TGGGGAATATTGGACAATGGA	Bacteria	Fusobacteriota	Fusobacteriia	Fusobacteriales	Fusobacteriaceae	Fusobacterium	gastrosuis	Fusobacterium_gastrosuis
ASV00002_Moraxella_pluranimalium	5,489	5,246	1,046	5,367	0,767	0,918	TGGGGAATATTGGACAATGGG	Bacteria	Proteobacteria	Gammaproteobacteria	Pseudomonadales	Moraxellaceae	Moraxella	pluranimalium	Moraxella_pluranimalium
ASV00059_Streptococcus_suis	0,212	0,129	1,646	0,170	0,780	0,925	TAGGGAATCTTCGGCAATGGG	Bacteria	Firmicutes	Bacilli	Lactobacillales	Streptococcaceae	Streptococcus	suis	Streptococcus_suis
ASV00198_Prevotella_Unclassified	0,146	0,142	1,026	0,144	0,806	0,950	TGAGGAATATTGGTCAATGGG	Bacteria	Bacteroidota	Bacteroidia	Bacteroidales	Prevotellaceae	Prevotella	Unclassified	Prevotella_Unclassified
ASV00140_Porphyromonas_Unclassified	0,234	0,173	1,358	0,203	0,815	0,951	TGAGGAATATTGGTCAATGGG	Bacteria	Bacteroidota	Bacteroidia	Bacteroidales	Porphyromonadaceae	Porphyromonas	Unclassified	Porphyromonas_Unclassified
ASV00155_Gemella_Unclassified	0,252	0,090	2,792	0,171	0,818	0,951	TAGGGAATCTTCCGCAATGGA	Bacteria	Firmicutes	Bacilli	Staphylococcales	Gemellaceae	Gemella	Unclassified	Gemella_Unclassified
ASV00093_Acinetobacter_Unclassified	0,734	0,450	1,631	0,592	0,825	0,952	TGGGGAATATTGGACAATGGG	Bacteria	Proteobacteria	Gammaproteobacteria	Pseudomonadales	Moraxellaceae	Acinetobacter	Unclassified	Acinetobacter_Unclassified
ASV00058_Actinobacillus_porcinus	1,175	0,475	2,475	0,825	0,855	0,980	TGGGGAATATTGCACAATGGG	Bacteria	Proteobacteria	Gammaproteobacteria	Pasteurellales	Pasteurellaceae	Actinobacillus	porcinus	Actinobacillus_porcinus
ASV00067_Pelistega_Unclassified	0,549	0,565	0,973	0,557	0,865	0,980	TGGGGAATTTTGGACAATGGG	Bacteria	Proteobacteria	Gammaproteobacteria	Burkholderiales	Alcaligenaceae	Pelistega	Unclassified	Pelistega_Unclassified
ASV00110_Unclassified_Unclassified	0,465	0,350	1,329	0,407	0,867	0,980	TGGGGAATTTTGGACAATGGG	Bacteria	Proteobacteria	Gammaproteobacteria	Burkholderiales	Neisseriaceae	Unclassified	Unclassified	Unclassified_Unclassified
ASV00162_Alysiella_Unclassified	0,178	0,189	0,944	0,183	0,877	0,984	TGGGGAATTTTGGACAATGGG	Bacteria	Proteobacteria	Gammaproteobacteria	Burkholderiales	Neisseriaceae	Alysiella	Unclassified	Alysiella_Unclassified
ASV00037_Neisseria_Unclassified	1,064	0,928	1,146	0,996	0,902	0,984	TGGGGAATTTTGGACAATGGG	Bacteria	Proteobacteria	Gammaproteobacteria	Burkholderiales	Neisseriaceae	Neisseria	Unclassified	Neisseria_Unclassified
ASV00192_Leptotrichia_Unclassified	0,169	0,118	1,429	0,144	0,903	0,984	TGGGGAATATTGGACAATGGG	Bacteria	Fusobacteriota	Fusobacteriia	Fusobacteriales	Leptotrichiaceae	Leptotrichia	Unclassified	Leptotrichia_Unclassified
ASV00068_Streptococcus_porcorum	0,847	0,848	0,999	0,848	0,905	0,984	TAGGGAATCTTCGGCAATGGG	Bacteria	Firmicutes	Bacilli	Lactobacillales	Streptococcaceae	Streptococcus	porcorum	Streptococcus_porcorum
ASV00083_Rothia_nasimurium	0,287	0,652	0,440	0,469	0,906	0,984	TGGGGAATATTGCACAATGGG	Bacteria	Actinobacteriota	Actinobacteria	Micrococcales	Micrococcaceae	Rothia	nasimurium	Rothia_nasimurium
ASV00240_Porphyromonas_Unclassified	0,159	0,142	1,120	0,150	0,908	0,984	TGAGGAATATTGGTCAATGGG	Bacteria	Bacteroidota	Bacteroidia	Bacteroidales	Porphyromonadaceae	Porphyromonas	Unclassified	Porphyromonas_Unclassified
ASV00244_Actinobacillus_indolicus	0,160	0,163	0,981	0,162	0,917	0,984	TGGGGAATATTGCACAATGGG	Bacteria	Proteobacteria	Gammaproteobacteria	Pasteurellales	Pasteurellaceae	Actinobacillus	indolicus	Actinobacillus_indolicus
ASV00035_Leptotrichia_Unclassified	1,256	0,843	1,490	1,049	0,920	0,984	TGGGGAATATTGGACAATGGG	Bacteria	Fusobacteriota	Fusobacteriia	Fusobacteriales	Leptotrichiaceae	Leptotrichia	Unclassified	Leptotrichia_Unclassified
ASV00144_Proteocatella_Unclassified	0,262	0,173	1,517	0,217	0,928	0,984	TGGGGAATATTGCACAATGGG	Bacteria	Firmicutes	Clostridia	Peptostreptococcales-Tissierellales	Peptostreptococcaceae	Proteocatella	Unclassified	Proteocatella_Unclassified
ASV00111_Prevotella_Unclassified	0,170	0,246	0,690	0,208	0,936	0,984	TGAGGAATATTGGTCAATGGG	Bacteria	Bacteroidota	Bacteroidia	Bacteroidales	Prevotellaceae	Prevotella	Unclassified	Prevotella_Unclassified
ASV00097_Porphyromonas_Unclassified	0,393	0,419	0,937	0,406	0,940	0,984	TGAGGAATATTGGTCAATGGG	Bacteria	Bacteroidota	Bacteroidia	Bacteroidales	Porphyromonadaceae	Porphyromonas	Unclassified	Porphyromonas_Unclassified
ASV00105_Porphyromonas_Unclassified	0,352	0,348	1,014	0,350	0,942	0,984	TGAGGAATATTGGTCAATGGG	Bacteria	Bacteroidota	Bacteroidia	Bacteroidales	Porphyromonadaceae	Porphyromonas	Unclassified	Porphyromonas_Unclassified
ASV00112_Actinobacillus_minor/porcitonsillarum	0,206	0,184	1,120	0,195	0,957	0,993	TGGGGAATATTGCACAATGGG	Bacteria	Proteobacteria	Gammaproteobacteria	Pasteurellales	Pasteurellaceae	Actinobacillus	minor/porcitonsillarum	Actinobacillus_minor/porcitonsillarum
ASV00009_Actinobacillus_porcinus	2,246	2,626	0,855	2,436	0,965	0,995	TGGGGAATATTGCACAATGGG	Bacteria	Proteobacteria	Gammaproteobacteria	Pasteurellales	Pasteurellaceae	Actinobacillus	porcinus	Actinobacillus_porcinus
ASV00044_Actinobacillus_minor	1,072	0,807	1,328	0,940	0,973	0,996	TGGGGAATATTGCACAATGGG	Bacteria	Proteobacteria	Gammaproteobacteria	Pasteurellales	Pasteurellaceae	Actinobacillus	minor	Actinobacillus_minor
ASV00050_Leptotrichia_buccalis	0,652	0,475	1,372	0,564	0,988	0,996	TGGGGAATATTGGACAATGGG	Bacteria	Fusobacteriota	Fusobacteriia	Fusobacteriales	Leptotrichiaceae	Leptotrichia	buccalis	Leptotrichia_buccalis
ASV00005_Moraxella_Unclassified	0,229	0,933	0,246	0,581	0,988	0,996	TGGGGAATATTGGACAATGGG	Bacteria	Proteobacteria	Gammaproteobacteria	Pseudomonadales	Moraxellaceae	Moraxella	Unclassified	Moraxella_Unclassified
ASV00114_Porphyromonas_Unclassified	0,536	0,405	1,323	0,470	0,993	0,996	TGAGGAATATTGGTCAATGGG	Bacteria	Bacteroidota	Bacteroidia	Bacteroidales	Porphyromonadaceae	Porphyromonas	Unclassified	Porphyromonas_Unclassified
ASV00072_Actinobacillus_indolicus	0,422	0,411	1,027	0,417	0,996	0,996	TGGGGAATATTGCACAATGGG	Bacteria	Proteobacteria	Gammaproteobacteria	Pasteurellales	Pasteurellaceae	Actinobacillus	indolicus	Actinobacillus_indolicus

Supplementary Figure S1.

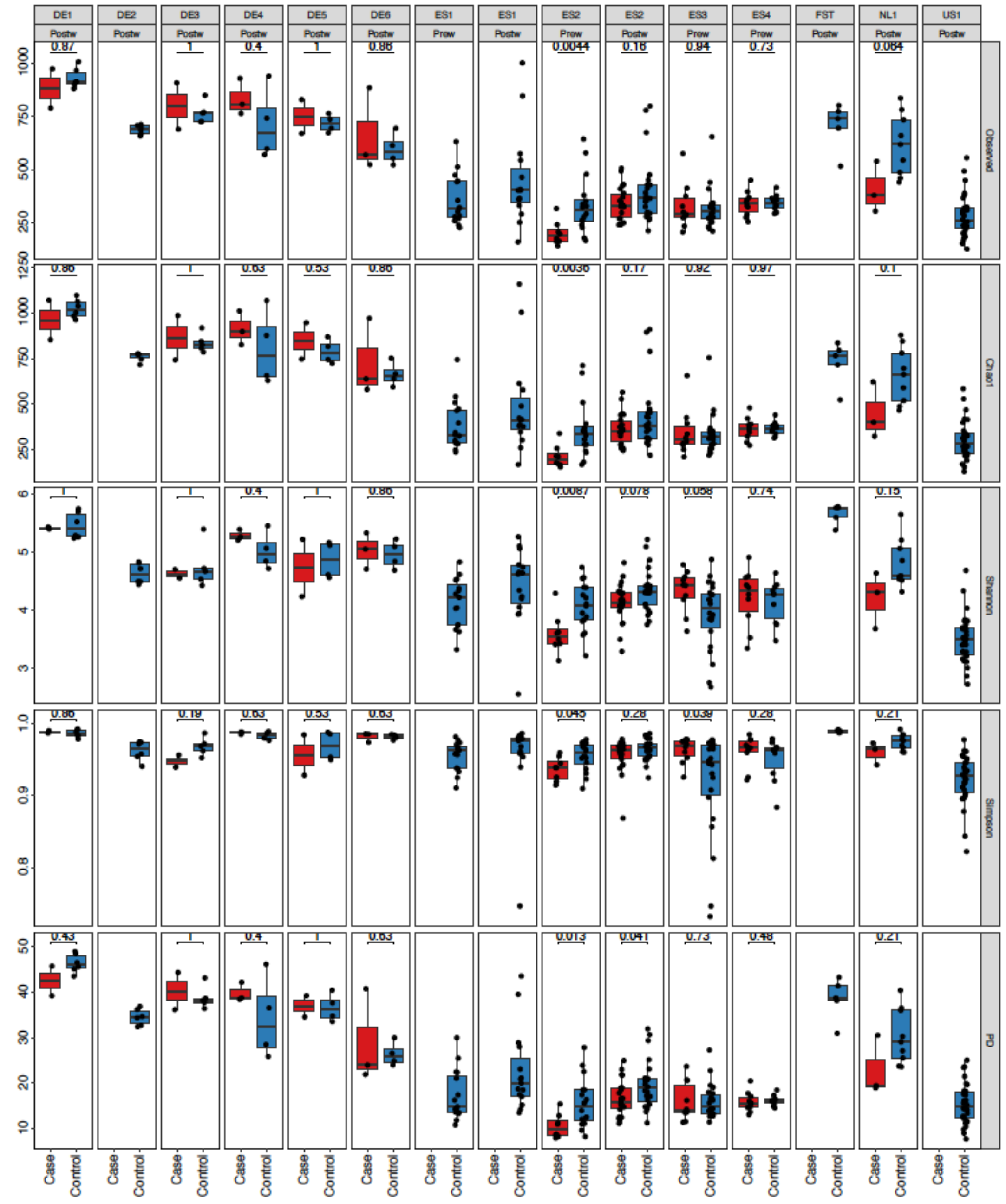

Figure S1: Comparison of alpha diversity in case and control piglets within each farm and age group. Plots based on different diversity metrics are shown facet on the y axis. The figure is based on amplicon sequencing data. Observed = the total number of ASVS detected in each sample. PD = phylogenetic diversity.
